# Risk Factors for Repetition of Self-Harm: A Systematic Review of Prospective Hospital-Based Studies

**DOI:** 10.1371/journal.pone.0084282

**Published:** 2014-01-20

**Authors:** Celine Larkin, Zelda Di Blasi, Ella Arensman

**Affiliations:** 1 National Suicide Research Foundation, Cork, Ireland; 2 School of Applied Psychology, University College Cork, Cork, Ireland; National Taiwan University, College of Medicine, Taiwan

## Abstract

**Background:**

Self-harm entails high costs to individuals and society in terms of suicide risk, morbidity and healthcare expenditure. Repetition of self-harm confers yet higher risk of suicide and risk assessment of self-harm patients forms a key component of the health care management of self-harm patients. To date, there has been no systematic review published which synthesises the extensive evidence on risk factors for repetition.

**Objective:**

This review is intended to identify risk factors for prospective repetition of self-harm after an index self-harm presentation, irrespective of suicidal intent.

**Data sources:**

PubMed, PsychInfo and Scirus were used to search for relevant publications. We included cohort studies which examining factors associated with prospective repetition among those presenting with self-harm to emergency departments. Journal articles, abstracts, letters and theses in any language published up to June 2012 were considered. Studies were quality-assessed and synthesised in narrative form.

**Results:**

A total of 129 studies, including 329,001 participants, met our inclusion criteria. Some factors were studied extensively and were found to have a consistent association with repetition. These included previous self-harm, personality disorder, hopelessness, history of psychiatric treatment, schizophrenia, alcohol abuse/dependence, drug abuse/dependence, and living alone. However, the sensitivity values of these measures varied greatly across studies. Psychological risk factors and protective factors have been relatively under-researched but show emerging associations with repetition. Composite risk scales tended to have high sensitivity but poor specificity.

**Conclusions:**

Many risk factors for repetition of self-harm match risk factors for initiation of self-harm, but the most consistent evidence for increased risk of repetition comes from long-standing psychosocial vulnerabilities, rather than characteristics of an index episode. The current review will enhance prediction of self-harm and assist in the efficient allocation of intervention resources.

## Introduction

Suicide is a significant health problem worldwide, with up to one million lives being lost to suicide annually [1]. Non-fatal deliberate self-harm is yet more prevalent and is associated with increased risk of suicide [2]–[4] and high costs in terms of health service resource utilisation [5]. Repetition of self-harm is common, particularly in the first weeks after an index hospital presentation of self-harm [6], [7]. The individual and societal costs associated with self-harm escalate with repetition: those who repeat self-harm are more than twice as likely to die by suicide compared with those who had engaged in self-harm n one occasion only [8]. Health service costs also increase with repetition [9] and repetition is indicative of persistent distress.

The effective prevention of self-harm requires multi-level intervention, ranging from community-based mental health promotion campaigns to clinical interventions with high-risk individuals [10]. However, accurate identification of individuals at risk of future self-harm is challenging. Extant research suggests that one of the strongest predictors of future self-harm is previous self-harm, but there is no perfect relationship between previous and future self-harm. One systematic review [4] reported that a median of 16% of self-harm patients repeat within one year, with the implication that presenting with self-harm in itself is an inadequate predictor of future self-harm.

With increasing constraints on acute hospital resources, those conducting risk assessments of self-harm patients could benefit from information on additional risk factors for future self-harm so as to effectively allocate resources to those most at risk. Indeed, risk assessment forms part of the recommended care for those presenting to emergency departments with self-harm. UK guidelines specify that risk assessment should include “identification of the main clinical and demographic features known to be associated with risk of further self-harm and/or suicide, and identification of the key psychological characteristics associated with risk, in particular depression, hopelessness and continuing suicidal intent” [11]. Unfortunately, this guidance is not sufficiently detailed and no recently published review exists which offers a comprehensive overview of risk factors for repetition of self-harm among self-harm patients. Similar reviews are out-dated [12], limited to non-suicidal self-injury [13], limited to psychometric assessment tools [14], or limited to examining one risk factor [15].

### Study Aims

The current systematic review is a synthesis of extant research on risk factors for repetition of self-harm among those presenting to emergency departments with self-harm. The purpose of this review to distil a burgeoning field into a digestible format for those conducting risk assessments of self-harm patients and to identify risk factors that are consistently associated with self-harm repetition, as well as identifying under-researched factors that show emerging associations with repetition.

## Methods

### Eligibility Criteria

Deliberate self-harm used was defined in accordance with the definition used in the WHO/EURO study, namely “an act with non-fatal outcome, in which an individual deliberately initiates a non-habitual behaviour that, without intervention from others, will cause self-harm, or deliberately ingests a substance in excess of the prescribed or generally recognized therapeutic dosage, and which is aimed at realising changes which the subject desired via the actual or expected physical consequences” [16]. It does not assume or preclude suicidal intent, and as such, encompasses self-harm acts where suicide intent is present (often referred to as “suicide attempts” or “parasuicide”) and where it is absent (often referred to as “non-suicidal self-injury”). This definition is used because of its inclusiveness and because it is one of the most widely used definitions of self-harm in the international literature. Language of publication did not form an exclusion criterion.

### Types of Studies

Because of the focus on prediction over time, studies were included if they adopted a longitudinal study design and were excluded if they adopted a cross-sectional design.

### Participants

As this review is intended to inform health professionals who conduct risk assessments with patients presenting with self-harm, we included only hospital-based studies, which recruited self-harm patients after they had presented to hospital with self-harm and which measured potential risk factors soon after presentation. The selected studies were those that compared factors between repeaters and non-repeaters.

### Outcome Measures

Studies were included if an outcome measure was prospective repetition of self-harm, either self-reported or derived from hospital records, over any length of follow-up. Both approaches to detecting repetition are of value: self-report is subject to report bias but is effective in detecting “hidden” self-harm whereas hospital records are less prone to report bias but limited to those who present to observed hospitals.

### Interventions

Studies were excluded if they were part of an intervention study, except in cases where the study involved only patients from the control arm.

### Search Strategy

MeSH was used to generate synonyms for deliberate self-harm (DSH). We searched for articles containing the following terms: synonyms for DSH (e.g., “self-harm”, “attempted suicide”, “parasuicide”, “self-injur*”, “self-poison*”), synonyms for repetition (e.g., “repeat*”, “recur*”, “re-present*”, “recidiv*”), and synonyms for cohort study (e.g., “follow-up”, “retrospective”, “predict*”, “prospective”, “longitudinal”). Journal articles, abstracts, letters and theses published in all years up to June 2012 were included. A literature search was conducted using the following databases: Scirus (up to June 2012), PubMed (up to June 2012), and PsycInfo (up to June 2012). For example, using the following identified 640 records in PsycInfo: (“self-harm*” OR “attempted suicide” OR “suicide attempt*” OR “self-injur*” OR “parasuicide*” OR “suicidal” OR “self-poison*” OR “self-cut*”) AND (re-present* OR repeat* OR repetition OR recur* OR recidiv*) AND (cohort OR longitudinal OR “follow-up” OR “followed up” OR prospective OR predict*) in “alltext” with no limits. A protocol for the current review was not pre-registered.

### Data Collection

A doctoral researcher used forms to extract the following variables from each located article: authors and year of publication; setting; location; eligibility criteria for case inclusion (suicidal intent: methods of self-harm; admission status); recruitment process; response rate; baseline number of participants; factors measured and operationalization used; duration of follow-up; means of repetition detection; retention rate; statistical methods used. In order to create crosstabs of certain variables and repetition for forest plots, papers were later revisited and corresponding authors were contacted by email. If the corresponding author did not respond, other authors were contacted by email.

### Quality Assessment

The risk of bias of each of the included studies was assessed using the original instrument outlined in Table 1. The selection of quality criteria for the instrument was based on key methodological concerns of extant checklists/guidelines [17], [18] while being tailored to cohort studies of self-harm patients. In line with a systematic review of existing quality assessment tools [17], our original instrument incorporates items for five core quality concerns including selecting study participants, measuring outcomes, addressing design-specific sources of bias, control of confounding and analysing data [19]. The cut-off points adopted in the instrument are based on typical recruitment/retention rates and means of repetition detection in published cohort studies of self-harm patients. The cut-off for adequate power (n = 175) is the minimum number of participants required to detect a small-medium Cohen's d of 0.3 in a two-tailed t test of independent samples with alpha level of 0.05.

**Table 1:** Quality Assessment Tool Used to Assess Located Studies Including Scoring Criteria.

Criterion	Scoring
Representativeness	1: Random/consecutive and response rate>70%
	0.5: Restrictive inclusion/exclusion criteria or response rate<70%
	0: Convenience sampling
Adequate power	1: Describes power calculations and was adequately powered
	0.5: Does not describe calculations but is adequately powered (n>175)
	0: Is not adequately powered
Appropriate outcome measure	1: Both self-report and hospital records
	0.5: Hospital records or self-report with ≤20% attrition
	0: Self-report with >20% attrition
Controlling for confounder variables	1: Confounders controlled for by design or statistical analysis
	0: Confounders not controlled for
Appropriate statistical analyses	1: Appropriate statistical analyses used
	0.5: Appropriate statistical analyses used in univariate or multivariate analyses only
	0: Appropriate statistical analyses not used

Strong evidence for the identification of a risk factor would be derived from a study which included all presentations of self-harm, which reported sample size calculations and was adequately powered, which used both self-report and hospital records to detect repetition, which controlled for confounders and which used appropriate statistical analyses. The score obtained using this tool is not necessarily a reflection of the quality of the study overall but rather the evidence that a particular risk factor is associated with repetition. For example, a well-designed study of adolescents would receive a score of 0.5 on “sampling” because the study excluded those who were not adolescent. Moreover, a number of studies [8], [20] did not focus on repetition as an outcome but as a factor associated with an alternative variable. These studies included multivariate analyses in predicting other variables but not repetition and they would receive a mark of zero for “controlling for confounder variables”.

### Synthesis of included studies

Given the multitude of risk factors investigated in the included studies, most of the associations between risk factors and repetition are presented in narrative form. The findings are arranged by estimated size of the association and number of relevant studies examining the specific risk factor (fewer than four, four to twelve, and more than 12). These arbitrary cut-offs are for the purposes of illustration only. Where counts of exposure and outcome were reported, odds ratios were calculated and used to inform the narrative; otherwise, we interpreted the original reported effect sizes, namely hazard ratios (HR), odds ratios (OR) and relative risk (RR) for dichotomous variables. For continuous variables, Cohen's d was calculated where possible.

Several factors that they had been examined extensively and appeared to show some consistency in their association with repetition are illustrated with forest plots. For each study that included these selected risk factors, the sensitivity and specificity of each factor in predicting repetition were calculated where possible. Forest plots of the values were generated using Review Manager 5.1 [21]. Some included studies reported that the association between one of these factors and repetition had been examined but did not report counts of exposure and outcome measures. In these cases, authors were contacted to obtain count data and these studies were excluded from forest plots if data was not made available. Two forest plots are included here for illustration (previous self-harm; personality disorder), while five others (previous psychiatric treatment; schizophrenia; alcohol abuse/dependence; drug abuse/dependence; living alone) are provided as Supporting Information.

Pooled estimates of predictive values were not calculated because of the methodological heterogeneity of the studies. The definition of a positive test varied among studies and often depended on judgment rather than measurement [22]. In addition, length of follow-up, which can affect the predictive power of a risk factor [23] varied between studies. Moreover, the results also appeared to be statistically heterogeneous: visual examination of the sensitivity and specificity values showed that they were extremely variable and therefore unsuited to meta-analysis.

## Results

### Characteristics of Located Studies

**Figure 1:**
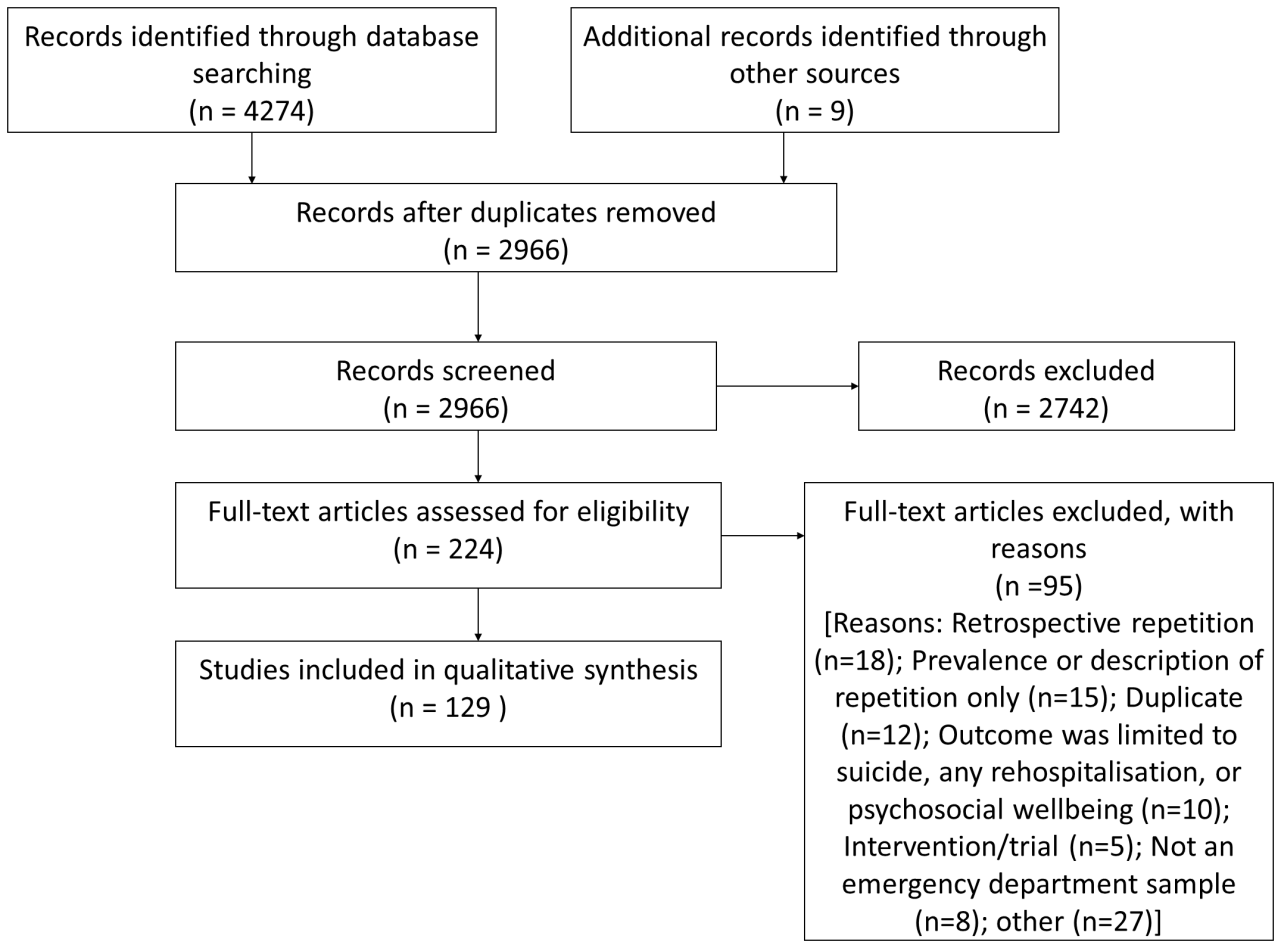
Flowchart of included studies.

The systematic literature search located 129 studies (Figure 1) involving a total of 329,001 index presentations, with some overlap between cohorts. The number of baseline participants ranged from 22 to 5,0891, with 23 (18%) studies involving less than 100 patients, 64 (50%) with between 100 and 1000 patients and 42 (33%) involving more than 1000 patients. The majority of included studies were conducted in Europe (106/129; 82%); 56 studies (43%) were conducted in the UK. Out of the 23 remaining studies, nine were conducted in Australia or New Zealand, eight were conducted in the US or Canada, two in India and one study each in Fiji, Hong Kong, Nicaragua, and Kuwait. One study was published in the 1960s, nine in the 1970s, 11 in the 1980s, 37 in the 1990s, 52 in the 2000s and 20 since 2010. In terms of the level of suicidal intent of the self-harm episodes, no studies were identified which included only non-suicidal self-injury, 11 studies included only patients who confirmed that their self-harm was intended to cause death, seven studies did not provide adequate information on intent, and the remainder (111/129; 86.0%) of the studies included self-harm of all levels of suicidal intent. Duration of follow-up ranged from three months to 41 years, with the most common follow-up period being 12 months. Given that cohort study design was one of the inclusion criteria of the review, the design of the studies were similar but the quality of the procedures of data collection and analyses varied. Using the scale outlined above, the mean quality score for studies was 3.0 out of a maximum score of 5. Forty-four studies scored up to and including 2.5 (low), 55 studies scored 3-3.5 (medium), and 30 studies scored 4 or over (high). The frequencies of scores for each of the five quality criteria are summarised in Table 2. The majority of the studies succeeded in recruiting a representative sample and in conducting appropriate statistical analyses. Many studies were underpowered but the larger studies tended to rely on only hospital records to detect repetition. About half of the studies controlled for confounding and such analyses were more common in publications from recent years.

**Table 2:** Frequencies of Each Score on Quality Assessment Tool of Located Studies (n = 129).

		Score	
Criterion	0	0.5	1
Representativeness	1	54	74
Adequate power	52	75	2
Appropriate outcome measure	19	99	11
Controlling for confounding variables	59	-	70
Appropriate statistical tests	6	0	123

This section will report on individual risk factors, reporting the extent to which they were studied and the magnitude of their associations with repetition, and end with an overview of composite predictive scales. For risk factors with a consistent association with repetition, forest plots were compiled to illustrate the sensitivity and specificity values of a risk factor across multiple studies. The characteristics of the included studies presented in “Supporting Information” in Table S1.

### Widely researched factors with moderate association with repetition

There were several factors that consistently showed medium-sized associations with repetition across numerous studies. The most consistent of these were previous self-harm; personality disorder; hopelessness; history of psychiatric treatment; schizophrenia; alcohol abuse/dependence; drug abuse/dependence; and living alone.

**Figure 2:**
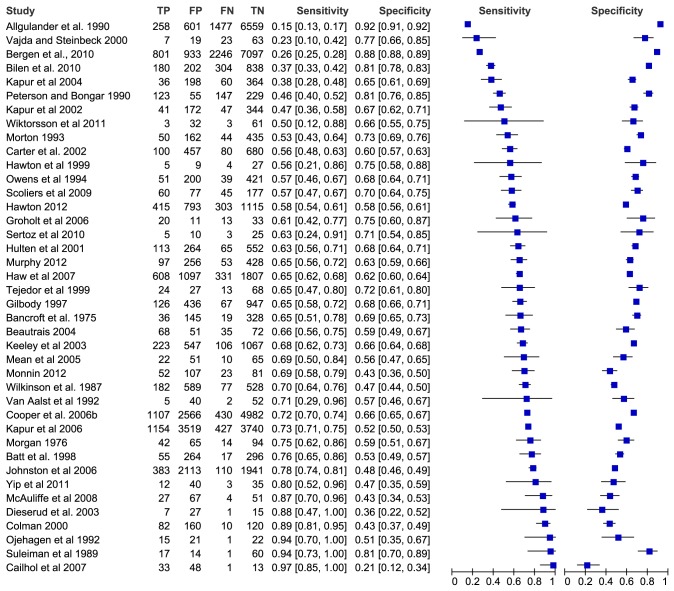
Forest plot of sensitivity and specificity of previous self-harm in predicting repetition.

Sixty of the eligible studies (47%) examined the association between previous self-harm and repetition. Using univariate analyses, 43 studies reported a significantly higher risk of repetition associated with previous self-harm, of which 30 were high-/medium-quality. Most odds ratios fell above 2.0. Seven studies reported a positive association between previous and subsequent self-harm that did not reach statistical significance. Furthermore, a significant positive relationship between previous self-harm and repetition was found in multivariate analyses in 20 high-/medium-quality studies, with effect sizes becoming slightly attenuated. Taken as a whole, these studies provide exceptionally consistent evidence of a medium-sized association between previous self-harm and repetition. The sensitivity and specificity of previous self-harm in predicting repetition were calculated where possible and are presented in Figure 2.

**Figure 3:**
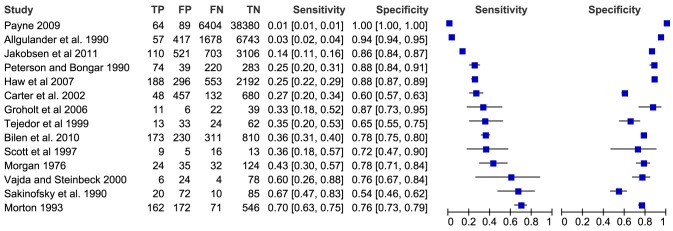
Forest plot of sensitivity and specificity of personality disorder in predicting repetition.

Seventeen studies examined the association between having a personality disorder (most often diagnosed using the ICD or DSM and documented in hospital notes) and repeating self-harm. Thirteen studies, twelve of which were high-/medium-quality, reported a statistically significant association, with univariate odds ratios ranging from 1.7 (95% CI: 1.4–2.0) [24] to 4.88 (95% CI: 1.27–18.72) [25] and exceptionally high odds ratio in one study of economically active men aged 16–64 years (OR = 7.25, 95% CI: 5.22–10.05) [26]. In contrast, two high-/medium-quality studies and two low-quality studies found no statistically significant association. Taken as a whole, these studies provide evidence of a relatively consistent and large association between personality disorders and repetition. The values for sensitivity and specificity of personality disorder in predicting repetition in individual studies were calculated where possible and are presented in Figure 3. The studies demonstrated a large variation in sensitivity from 0.01 to 0.70, but specificity was better with a lowest value of 0.63.

Twenty-five studies (19%) examined hopelessness as a predictor of DSH repetition. One systematic review [15] examining whether a high score (≥9) on the Beck Hopelessness Scale could predict DSH located six studies, four of which were conducted in emergency department settings; three studies meet the inclusion of the current review [20], [23], [27] and one does not because it involved an intervention [28]. McMillan and colleagues found that using a cut-off point of 9 gave a pooled sensitivity of 0.77 (95%CI: 0.72–0.81) and pooled specificity of 0.41 (95% CI: 0.37–0.45) with a pooled diagnostic odds ratio of 2.27 (95% CI: 1.53–3.37). They concluded that the low specificity of the BHS in predicting repetition precludes the use of the BHS as a tool to allocate treatment. In the time since the systematic review was conducted, two further high-medium quality studies examined the association between high BHS scores and repetition of DSH [29], [30]. Both found no statistically significant difference in repetition risk associated with scoring over 14, with odds ratios of 1.90 and 1.41 respectively. Thirteen studies used the BHS as a continuous variable in univariate analyses, of which eight studies reported significantly higher scores in repeaters than non-repeaters (differences in mean scores between repeaters and non-repeaters ranged from 1.0 to 4.9 points). Three high-/medium-quality studies using multivariate models reported increased odds of repetition that were not statistically significant. Overall, these findings indicate a consistent moderate association between BHS scores and repetition.

History of psychiatric treatment has been examined in association with repetition in 29 studies (22%). Twenty-two studies reported a significantly increased risk of repetition associated with having a history of psychiatric treatment, of which 19 were high-/medium-quality and three were lower quality. Odds ratios fell around 3.00. Seven studies found no significant association, of which four were high-/medium-quality and three were lower quality. However, those studies reporting effect sizes indicated that the effects were in the positive direction. Overall, these findings indicate a consistent and sizeable association between repetition and a history of psychiatric treatment. The sensitivity values of the factor were extremely variable and specificity values tended to be moderate (Figure S1).

Twenty-one studies (16%) examined the association between schizophrenia and repetition, of which 19 reported increased odds of repetition in this group (with odds ratios ranging from 1.24 to 7.76). However, the association only reached statistical significance in eight studies, most likely reflecting the relative rarity of this diagnosis. Reflecting the relative rarity of the diagnosis, sensitivity values were very low but specificity values tended to be close to 1.00 (Figure S2).

Alcohol abuse/dependence was examined in 33 (26%) studies. Nineteen high-/medium-quality studies reported an increased risk of repetition associated with alcohol abuse/dependence, with most odds ratios close to 2.00. Fourteen additional studies (of which nine were high-/medium-quality) found no statistically association between alcohol abuse/dependence and repetition, although the associations tended to be in positive direction. Such evidence demonstrates a relatively consistent and moderate association between repetition and alcohol abuse/dependence. Sensitivity values were consistently low, mostly around 0.2, while specificity values mostly fell around 0.80 (Figure S3).

Drug abuse/dependence was examined in 23 (18%) studies. Thirteen high-/medium-quality studies reported a positive association between repetition and drug abuse/dependence with slightly larger effect sizes than those seen in studies of alcohol dependence. Ten studies found no statistically significant association, of which eight were high-/medium-quality and two were lower quality. Taken as a whole, these studies suggest there is a moderately increased risk of repetition among those with drug misuse/dependence and repetition.. Like alcohol misuse, the sensitivity values for drug misuse were low, with most of the values falling around 0.20, whereas specificity tended to be very high (Figure S4).

Eighteen studies (14%) examined the association between repetition and living alone. Most of these studies reported that those who live alone are more likely to repeat self-harm, with positive odds ratios ranging from 1.06 to 3.28, and most falling around 1.5. Five studies conducted multivariate analyses: the association persisted in one study [31] but not in the other four studies [2], [32]–[34]. These studies showed a fairly consistent moderate-sized association between living alone and repeating self-harm. The values for sensitivity of living alone in detecting repetition risk were fairly consistently moderate, with corresponding high values for specificity (Figure S5).

### Less often-researched factors with moderate associations with repetition

There were several other factors that were less often-researched (examined in 4–12 studies) but showed emerging evidence of a moderate association with repetition, namely impulsivity; comorbidity; problem-solving ability; sexual abuse; current psychiatric treatment; stressful life events; work or school problems (protective); relationship problems (protective); family relationship problems; financial problems (protective); attitude towards self-harm episode; and involvement of self-cutting.

Seven studies examined the association between impulsivity and repetition. Four high-/medium-quality studies used the Barratt Impulsivity Scale (BIS) and, using a variety of cut-off scores, all found a positive small to medium associations with repetition. Five studies used a variety of impulsivity measures and found higher scores in repeaters. Comorbidity was associated with a significantly higher risk of repetition in five of six studies. Limited problem-solving ability and negative problem-solving skills were associated with higher repetition risk in four high- to medium-quality studies. Six high-/medium-quality studies and three lower-quality studies reported increased risk of repetition among those who had been sexually abused with odds ratios ranging from 1.4 to 7.2, with all but one association reaching statistical significance. In three studies of adolescents (which also were three studies that included only those with a confirmed intention to die), a history of sexual abuse was not found to have a statistically significant association with repetition.

Current psychiatric treatment at the time of the index episode was examined in ten studies. It was significantly associated with an increased risk of repetition in nine high-/medium-quality studies and had no association with repetition in one lower-quality study, with odds ratios failing around 2.5. Reporting stressful life evens was associated with a significantly higher risk of repetition in three high-/medium-quality studies and two lower quality studies, and had no association in two studies. Current work or school problems had a medium-sized protective effect against repetition in four high-/medium quality studies with odds ratios from 0.52 to 0.74. Three lower quality studies [35]–[37] reported non-significant effects in both directions. Relationship problems had a moderate protective effect against repetition if the relationship in question was one with a partner (ORs: 0.53 to 0.64), friends (ORs: 0.66 to 0.90), or others (ORs: 0.64 to 0.90) but reporting problems with family relationships conferred a slightly increased risk of repetition (ORs: 1.02 to 2.91). Four high-/medium-quality studies found a moderate protective effect of financial problems on repetition [31], [33], [38], [39] with odds ratios ranging from 0.59 to 0.82, whereas four studies reported non-significant associations in both directions, of which two were high-/medium-quality [40], [41] and two were lower quality [36], [42]. Six studies (of which one was lower-quality) found significantly increased risk of repetition among those who responded with regret or anger to surviving, or who continued to have suicidal plans at the time of assessment. Six high-/medium-quality studies reported an increased risk of repetition associated with involvement of self-cutting in particular, with odds ratios ranging from 1.18 to 2.25.

### Under-researched factors with associations with repetition

There were many factors that had significant associations with self-harm repetition, but were only examined in three or fewer studies. These included self-efficacy; short-short genotype functional polymorphism in the serotonin transporter gene promoter region; epilepsy; compiling a will (protective); non-White ethnicity (protective); external hostility; frequent nightmares; being a victim of violence; having parents separated or divorced; dysfunctional family of origin; having affectionless controlling parents; bipolar disorder; post-traumatic stress disorder; Global Assessment of Functioning Scale score; homelessness; living in an institution; reporting self-harm as being a direct response to mental symptoms; and lower socioeconomic status.

### Factors with small associations with repetition

Several factors exhibited only small associations with repetition. Thirty-nine studies (30%) examined the association between repetition and marital status. Fifteen studies (of which thirteen were high-/medium-quality) found a significantly lower risk of repetition associated with being married or cohabiting (odds ratios ranged from 0.29 [36] to 0.80 [33]), 23 studies found no significant association between marital status and repetition, and one lower quality study reported a significantly increased risk of repetition associated with being married [43].

Fifty-eight studies (45%) examined the association between repetition and age. These studies indicate that those who go on to repeat tend to be younger than non-repeaters, except within adolescent groups where there is some evidence that older adolescents are at higher risk than younger adolescents. The relationship between unemployment and repetition of self-harm was explored in thirty-two studies (25%). Only nine studies, of which five were high-/medium-quality, found a significant positive association with moderate odds ratios using univariate analyses. These studies yield evidence of a small positive association between repetition and unemployment.

Factors that were examined in three or fewer studies and had a small effect on repetition risk were: area-level factors; history of violence/criminal record; sense of coherence; type of medication used in overdose; housing problems; frequent relocation; and involvement of self-injury; non-native nationality; obsessive-compulsive symptoms; trait anger; internal hostility; lower autobiographical memory specificity; alexithymia; neuroticism; sociopathy; higher platelet serotonin; 24-hour urinary cortisol levels; physical abuse; being separated from or bereaved of parents in childhood; poor familial economic circumstances; emotional abuse; mean number of adversities; antisocial disorder; being a pensioner/retired; suicidal thoughts at time of assessment; and non-verbal behaviour at the time of assessment.

### Contradictory evidence

Some factors were widely researched (i.e. examined in more than 12 studies) but generated contradictory evidence as to whether they were risk factors for repetition, namely gender; mood disorder; physical health problems; and suicidal intent.

There was contradictory evidence on how gender affected repetition risk, with small effects observed in both directions in 68 studies. Neither was mood disorder consistently associated with repetition risk, with only 8 of 24 studies reporting an effect, with sizeable odds ratios, ranging from 2.18 [44] to 6.19 [45]. Physical health problems had a sizeable positive association with repetition in two studies (ORs = 2.15 and 1.88), negative association in two studies, (ORs = 0.09 and 0.26), and no association in ten studies.

Of 16 studies examining the association between repetition and total Beck Suicide Intent Scale score, one high-/medium-quality and two lower-quality studies found a significant moderate negative association with repetition, with a larger effect for men (d = −0.43) than women (d = −0.15) in one study. Two high-/medium-quality studies [39], [46] found a negative association with repetition in males only. Eleven studies found no association with repetition, of which seven were high-/medium-quality. Effects tended to be small to moderate (d = −0.17 to +0.59) and were more often in a positive direction. These results indicate that the suicidal intent associated with an index episode is not a reliable predictor of repetition, but that gender should be explored as a potential moderator.

There were other factors examined in fewer studies for which there was contradictory evidence on their association with repetition, namely self-esteem [47] [25] [32], [48]; self-report general mental health measures [30], [35], [49]–[51]; pregnancy-related problems [24], [33], [38], [41]; perceived social support and loneliness [32], [35], [39], [41], [52]–[55]; involvement of major self-injury [24], [31], [34], [56]–[58]; borderline personality disorder [59]–[61]; dysthymic disorder [49]; and ingesting multiple drugs [62], [63].

### Factors with no evidence of an association

Level of education and lethality/medical seriousness of an index act were examined in many (>12) studies and consistently had no effect on repetition risk. Three high-/medium-quality studies found a significant protective effect of reaching a higher level of education with odds ratios ranging from 0.15 (95% CI: 0.03–0.81) to 0.70 (95% CI: 0.49–0.99). However, seven high-/medium-quality studies and three lower-quality studies found no statistically significant difference in education level between repeaters and non-repeaters, although the effect sizes suggest a very small protective effect. The association between repetition and lethality or medical seriousness was examined in 17 studies. One high-/medium-quality study found a sizeable positive association and three studies of varying quality found moderate inverse associations. The remainder of the studies reported non-significant effect sizes.

Some factors were examined in a moderate number (4–12) of studies and had no association with repetition, namely self-rated problem-solving ability; family history of suicide; anxiety disorders; adjustment disorder; depression scores; anxiety scores; legal problems; bereavement; having children; wish to die; premeditation; suicide note; steps to avoid discovery; involvement of alcohol; substance misuse and motives for the index episode.

Some factors were examined in only a fewer than four studies and had no association with repetition, namely the presence of an eating disorder; family psychiatric history; locus of control; interpersonal sensitivity; platelet monoamine oxidase activity; rate-limiting enzyme of serotonin synthesis; sleep problems; CSF 3-methoxy-4-hydroxyphenylglycol; CSF hydroxyindoleacetic acid; paroxetine binding; CSF homovanillic acid; post dexamethasone-suppression test plasma cortisol levels; premenstrual tension; quality of parental relationship and unhappy childhood; high emotional expression in the home; self-harm by a friend or relative; link to parents; organic mental/cognitive disorder; state anger; bullying; receiving state payments; isolation at the time of the act; undertaking final acts in preparation for death; and help-seeking during or after the index act.

### Composite Predictive Scales

Nine studies created or adapted scales specifically to identify those at high risk of repetition of self-harm. (Table 3). Such a tool should have good sensitivity and specificity and should be easy to administer in a busy emergency department setting. In the case of risk assessment, a false negative involves more severe consequences than a false positive, so sensitivity is particularly important when choosing scales to predict repetition.

**Table 3:** Frequencies of Each Score on Quality Assessment Tool of Located Studies (n = 129).

Scale	Items	Cut-off	Sensitivity	Specificity
Buglass and Horton scale [40]	Sociopathy; problem in the use of alcohol; previous psychiatric in-patient care; previous psychiatric out-patient care; previous parasuicide admission; and not living with a relative	Various	>80%	56%–67%
Edinburgh Risk of Repetition Scale [66]	Previous parasuicide, personality disorder, alcohol problems, previous psychiatric treatment, unemployment, social class, drug abuse, criminal record, violence (given or received), age and marital status	Clinical cut-off: 8 for males, 6 for females	17.1–33.3%	84.0–94.7%
Manchester self-harm rule [38]	History of self-harm, previous psychiatric treatment, benzodiazepine use in this attempt, any current psychiatric treatment.	Positive response to any item	94–97%	25–26%
Suicide Assessment Scale (SUAS) [71]	Sadness and despondency, tension, emotional withdrawal, perceived loss of control, and suicidal thoughts	24	61%	40%
SAD-PERSONS [73]	Sex, age, depression, previous attempt, ethanol abuse, rational thinking, social support, organised plan, no spouse, and sickness	5	Not reported	Not reported
Corcoran et al [74]	Any previous act of self-harm, main method of self-harm used, alcohol taken at time of act, drugs taken as part of act, change in domestic situation near time of act, history of abuse of street drugs, marital status, level of education, harm caused by alcohol, age, and sex	Three groups: Low (0–0.2); Medium (0.2–0.45) High (>0.45)	0.2 cut-off:96.15% 0.45 cut-off: 80.77%	0.2 cut-off: 81.4% 0.45 cut-off: 89.53%
Colman et al.[27], [32]	Prior history of self-harm, lifetime history of schizophrenia, lifetime history of depression, and fair or poor physical health over the preceding three months	2–3	73.9%	70.0%
Petrie and Brook [75]	Age, employment, sense of coherence subscales, living alone, previous attempts, method of self-harm, hopelessness, sex, marital status, self-esteem, depression	Not reported	63.2%.	67.9%
ReACT rule [31]	Recent self-harm (self-harm in the past year), living alone or homeless, present with self-cutting as a method of self-harm, treated for a current psychiatric disorder	4	95%	21%
Assessment for Repeated Suicide [76]	Items from well-established measures of hopelessness, impulsivity, aggression, and suicidal ideation	NA	NA	NA

#### Buglass and Horton scale

The Buglass and Horton scale [40] consists of six dichotomous items, the presence of each being assigned a value of one. The validity of the scale was examined in six subsequent studies. The sensitivity of the scale tends to be high, falling above 80%, but its specificity is medium, falling between 56% and 67% [12], [40], [42], [64]. Two studies used a score on the scale as a continuous variable and found significantly increased risk of repetition with higher scores [23], [65]. Scott et al.[52] found that having a score of greater than three on the scale was not more likely among repeaters than non-repeaters (OR = 1.13, 95% CI: 0.31–4.03), but the sample in that lower-quality study was limited to patients who received a score of one or higher on the scale.

#### Kreitman and Foster scale

Kreitman and Foster [66] developed the Edinburgh Risk of Repetition Scale, whose validity was examined in three further studies. The 11 item-scale developed in a high-/medium-quality study by Kreitman and Foster [66] was designed to be suitable for both clinical and research settings, with dichotomous responses in the former version and weighted responses in the latter. The authors used a further cohort to validate the scale and found high sensitivity using the clinical version, but low specificity. Three high-/medium-quality studies [67]–[69] explored the predictive value of this scale, with mediocre effects on repetition The sensitivity of the scale was mediocre and found that the research version outperformed the clinical version in one of the cohorts. The scale performed less well when repetition was calculated per full year as opposed to per calendar year and when repetitions were based on persons rather than admissions. The Buglass and Horton scale and Kreitman and Foster scale have been found to perform comparably [69].

#### Manchester self-harm rule

In a derivation set of 6,933 self-harm presentations, Cooper et al.[38] generated an optimal decision rule incorporating four dichotomous variables. A positive response to any of the four variables indicated risk, correctly identifying 94% of repeaters in the derivation set and 97% of repeaters in the validation set. This exceptionally high sensitivity, however, was accompanied by a low specificity (25% and 26% in the derivation set and validation set respectively). The rule additionally predicted all completed suicides. A further study using the same data showed that the sensitivity of the rule was superior to that of risk assessments by both mental health specialists and ED physicians [70]. Two more recent studies have demonstrated high sensitivity but low sensitivity [30], [31].

#### Suicide risk scales

In a high-/medium-quality study, Waern et al. [71] used the modified Suicide Assessment Scale (SUAS) to predict repetition of self-harm. The tool contains items rated on a five-point scale. Patients who obtained a high score on the SUAS (>30) were significantly more likely to repeat, even after adjustment for age, sex, anxiety and depression. A cut-off score of 24 optimised sensitivity and specificity, which were quite poor at 61% and 40% respectively. The SAD-PERSONS scale [72] was originally based on risk factors for suicide and has ten dichotomous items. A score of 0–4 indicates low risk and 5–10 indicates high risk. The scale was used in a lower quality study by Öjehagen et al. [73] to predict repetition of self-poisoning. They found no association between repetition and scores on the scale (d = 0.44).

#### Other predictive scales

The remaining scales have not yet been validated. Corcoran et al.[74] identified eleven predictor variables in a high-/medium-quality study. Three cut-off points can be adopted for acceptable sensitivity and specificity, high sensitivity, or high specificity, depending on the purposes of the investigation. The scale also allows for the classification of patients into low-, medium-, and high-risk groups.

In multivariate analyses in a high-/medium-quality study, Colman et al.[27], [32] identified four independent dichotomous risk factors for repetition. Giving equal weighting to each item, he concluded that a cut-off score of between two and three optimised sensitivity and specificity at 73.9% and 70.0% respectively. In a high-/medium-quality study, Petrie and Brook [75] conducted a discriminant analysis to investigate how a number of variables discriminated between repeaters and non-repeaters. The analysis indicated that the variables had a specificity of 67.9% and a sensitivity of 63.2%.

More recently the ReACT rule [31] has been developed including non-assessed presentations. It states that a person is at high to moderate risk of repetition if they have four specific risk factors. In the derivation data, the rule had 95% sensitivity and 21% specificity. The rule was tested in external test data from another site in the same study and showed decreased sensitivity (90%) but improved specificity (34%).

Yeh et al. [76] developed the Assessment for Repeated Suicide using a number of self-report items from well-established measures of hopelessness, impulsivity, aggression, and suicidal ideation. Repeaters scored more highly than non-repeaters, with a moderate to large effect size (d = 0.64). A logistic regression controlling for age and marital status showed a small but statistically significant association between total ARS scores and repetition (OR = 1.06, 95% CI = 1.03, 1.09).

In summary, several tools for the prediction of repetition, such as the Manchester Self-Harm Rule and the ReACT rule have high sensitivity but poor specificity, which may have implications for the effective allocation of resources to those at risk.

## Discussion

### Summary

This review synthesises studies examining risk factors for repetition of self-harm after an index hospital presentation of self-harm. We identified factors that had been examined in relationship to repetition of self-harm and found several factors that consistently had statistically significant associations with repetition, namely previous self-harm, personality disorder, hopelessness, history of psychiatric treatment, schizophrenia, alcohol abuse/dependence, drug abuse/dependence, and living alone. However, these factors demonstrated poor sensitivity of individual risk factors in predicting repetition. Our findings also suggest that extant scales for predicting repetition of self-harm generally have adequate sensitivity but poor specificity.

### Factors Associated with Repetition

Through a systematic search, we located 129 eligible studies. Several risk factors have been widely studied and demonstrated consistent associations with repetition. A stepwise increase in the number of previous self-harm episodes has been shown to be consistently associated with a higher risk of prospective repetition [39], [57]. Similarly, having greater number of psychiatric disorders was associated with a higher risk of repetition in the current review. Such findings suggest that self-harm repetition is related to a constellation of related vulnerabilities, which need to be assessed at the time of presentation. Psychiatric morbidity and treatment history are usually routinely assessed at presentation and may therefore be easily incorporated into risk assessments. Existing risk factor scales incorporate most of the consistent predictors identified in the current review. Three scales included previous psychiatric treatment [38], [40], [66], four included alcohol problems [40], [66], [73], [74], and most of the scales included previous self-harm as a risk factor [27], [31], [32], [38], [40], [66], [73]–[75]. Other factors were less widely researched but there is increasing evidence to support their association with increased risk of repetition, including impulsivity; comorbidity; problem-solving ability; sexual abuse; current psychiatric treatment; stressful life events; work or school problems (protective); relationship problems (protective); family relationship problems; financial problems (protective); attitude towards self-harm episode; and involvement of self-cutting.

### Methodological Considerations

There are a number of methodological concerns to bear in mind in the interpretation of this review. In spite of relatively narrow inclusion criteria, the included studies were heterogeneous in terms of the instruments used to measure risk factors, the duration of follow-up, and methods used to detect repetition.

While it is of value to form a broad overview of risk factors for repetition, future reviews could focus on specific follow-up periods or specific measures of risk factors. We presented sensitivity and specificity values for selected risk factors; while helpful in terms of investigating the predictive value of individual risk factors, this approach did not enable us to examine how various combinations of risk factors might result in redundancy or increased predictive value. In terms of external validity, the current review was limited to longitudinal/prospective studies of hospital presentations of self-harm. Therefore, the risk factors for future self-harm identified in the review may not be applicable to the prediction of future self-harm in non-clinical groups. This approach allowed us to inform risk assessments conducted in acute hospital settings but further work is required to elucidate risk factors for repetition of self-harm among the sizeable population of self-harmers that never comes to the attention of emergency health services. Although some of the studies included in the current review counted a small number of completed suicides in the “repeaters” group, we did not explicitly address risk factors for suicide among self-harm patients. Although fatal repetition is rarer than non-fatal repetition, it is nonetheless an important clinical outcome, and designating suicide cases as non-repeaters in the current review could have resulted in the underestimation of the effect of some risk factors. For example, suicidal intent was not consistently associated with repetition in the included studies, but suicidal intent is an important predictor of eventual suicide [77]. It should also be acknowledged than the current review does not address all factors that may be of use in assessing risk in self-harm patients, and that further research is required to synthesise extant research on risk factors for completed suicide. Moreover, a factor such as drug abuse, though there is not yet strong evidence for an association with repetition, may still be considered important in the context of a needs assessment. This review therefore does not include all the measures that may be of interest to those conducting a complete psychosocial assessment. Another potential limitation of the review is that the vast majority of studies were conducted in Europe, such that the findings may not be generalizable to developing country settings. It is unclear whether this uneven distribution of studies is a fair reflection of the international research agenda or an indication of inclusion criteria that favoured the inclusion of European studies.

### Clinical Implications

Clinical guidelines for the management of self-harm recommend a risk assessment as part of the assessment process [11]. However, to date, no review has been published that draws together the extensive research on risk factors for repetition in order to inform these risk assessments. The current review suggests a number of easily-measured risk factors which can be incorporated into risk assessments. Clinicians are recommended to use risk factors scales such as the Manchester Self-Harm Rule or the ReACT rule, as these have been found to incorporate some of the most consistent predictors. It must be borne in mind, however, that these factors have only a predictive and not necessarily a causal association with repetition. It is possible that the risk factors identified in the review do not confer an inherent risk for repetition but rather affect the likelihood that a person will receive an assessment or access the support and treatment they require. Patients who misuse alcohol, have personality disorders, or an extensive history of self-harm may be the very patients least likely to engage with services but they are also perhaps less likely to encounter a positive response from health service providers. Engaging in self-harm indicates distress and coping difficulties and all patients should be facilitated in a way that recognises and addresses their needs. The findings of this review can be used to identify who is at risk of repetition such that constrained resources can be directed to those most at risk. Moreover, the review can serve to dispel misconceptions around the association between some factors and repetition. For example we found that a patient who does not report a wish to die is just as prone to repetition as a patient who does report a wish to die. We can conclude that there are a number of factors that are consistently associated with repeated self-harm, that represent items that should be part of any clinical assessment tool used to assess risk of repeated self-harm. However, the variable performance of these factors in terms of their sensitivity underlines the difficulty in predicting repetition, and suggests that accurate prediction of repetition of self-harm remains a challenge.

### Theoretical Implications

Despite the wide variety of theoretical frameworks within the self-harm literature [78]–[84], there is a dearth of theories that attempt to explain why a minority of those who present with self-harm will go on to repeat self-harm. The predictors of repetition identified in the current review span psychiatric, psychological and social domains, but seem to echo the factors involved in the initiation of self-harm. It might be argued that those who repeat self-harm prospectively possess risk factors for self-harm initiation to a higher degree than those who do not repeat self-harm. This is particularly applicable to continuous factors such as hopelessness, problem-solving ability, self-efficacy, sense of coherence and serotonergic functioning. This suggests that repetition risk might be associated with pre-existing vulnerability, as well as being affected by self-harm consequences in terms of access to appropriate health and social services. Interestingly, certain stressors such as work/school problems and relationship problems were inversely associated with repetition. This is in line with the “suicidal process” model, which conceptualises suicidal behaviour as becoming increasing autonomous with repetition [85].

It is of note that depression, a state measure considered to be the “final common pathway leading to suicidal behaviour” [83], was not particularly useful in predicting repetition. It may be that differences between repeaters and non-repeaters emerge in the period after an index episode where those who go on to repeat continue to experience depressive symptoms for a longer period or experience recurring depression at a later time.

In spite of the association in the current review between diathesis factors and repetition, it is not necessarily true that stressors have no role to play. The current study was focussed on prediction of future self-harm at the time of an index episode. An alternative approach to explore the processes involved in repetition is to compare index episodes of self-harm to repeat episodes. This approach allows for the investigation of the association between repetition and life circumstances, such as psychosocial adversity. It is conceivable that those who go on to repeat self-harm are those who experience more psychosocial adversity in the period after presenting with an index episode of self-harm.

### Limitations

The current systematic review sought to synthesise the large body of evidence around risk factors for repetition of self-harm into a form that is useful for those conducting risk assessments of self-harm patients and identify risk factors that would be suitable for further investigation by researchers. Unfortunately, the scope of the review precluded the in-depth examination of individual risk factors, and the methodological heterogeneity of the studies precluded meta-analyses. Although language of publication did not form an exclusion criterion, it may be that papers published in other languages were not detected during the systematic search. In terms of the limitations of individual studies, most studies relied on repetition detection through hospital presentation only, and hence designated those who repeated without presenting to hospital and those who died by suicide as “non-repeaters”.

### Future Research

This review indicates a number of consistent, widely studied predictors of repetition of self-harm. Future research could ascertain the respective strength of each of these predictors using meta-analysis, following the approach adopted by McMillan et al. [15] to examine the utility of the Beck Hopelessness Scale in predicting repetition. The current review identifies a number of risk factors which have been less extensively studied but which have so far exhibited positive associations with self-harm repetition. These risk factors should be incorporated in future self-harm studies in order to verify their usefulness in predicting repetition. A sizeable portion of these factors are psychological measures, which are less often incorporated into larger studies of self-harm patients. However, the recording of one or two psychological variables in these larger studies could potentially enhance repetition prediction, theory, and psychological interventions. Further work is also required to validate existing risk assessment scales considering that, to date, only three of the located scales were validated in subsequent studies.

There exist a variety of interventions which aim to prevent repetition of self-harm [86] and, although there is evidence that these are effective, the mechanisms involved are not routinely examined. Another way to identify factors associated with repetition would be to identify active components within effective complex interventions to reduce repetition. Such process evaluations can build a case for causal associations and make for more cost-effective and targeted interventions, as well as suggesting risk factors for future research. Conversely, the risk factors generated by the current review could point to targets for intervention, both psychological (e.g., hopelessness, problem-solving) and social (e.g., homelessness, victimisation). Finally, studies which follow up self-harm patients in the time after an index episode have the opportunity to provide a more complete picture of participants' situations by focussing on other outcomes such as psychopathology, wellbeing, and psychosocial circumstances in addition to repetition of self-harm.

### Conclusion

This review located a substantial number of studies of risk factors for repetition, most of which were of moderate quality. It appears that the most consistent predictor of repetition of self-harm is a history of self-harm, but there are several other risk factors emerging from the literature, including personality disorder, hopelessness, history of psychiatric treatment, schizophrenia, alcohol abuse/dependence, drug abuse/dependence, and living alone. This review is intended to inform those who conduct risk assessments of self-harm patients but also to identify gaps in extant research so that the focus can move from the identification of individual risk factors to a more comprehensive theoretical account of self-harm repetition.

## Supporting Information

Figure S1 **Forest plot of sensitivity and specificity of previous psychiatric treatment in predicting repetition.**
(EPS)

Figure S2 **Forest plot of sensitivity and specificity of schizophrenia in predicting repetition.**
(EPS)

Figure S3 **Forest plot of sensitivity and specificity of alcohol misuse in predicting repetition.**
(EPS)

Figure S4 **Forest plot of sensitivity and specificity of drug misuse in predicting repetition.**
(EPS)

Figure S5 **Forest plot of sensitivity and specificity of living alone in predicting repetition.**
(EPS)

Table S1 **Characteristics of Included Studies of Self-Harm Repetition.**
(DOCX)

Checklist S1 **Preferred Reporting Items for Systematic Reviews and Meta-Analyses: The PRISMA Statement Checklist**
(DOC)
