# Correction: Risk Factors for Repetition of Self-Harm: A Systematic Review of Prospective Hospital-Based Studies

**DOI:** 10.1371/annotation/b3dfd1b3-ada1-4f7e-89d4-a25ddf5d204a

**Published:** 2014-01-28

**Authors:** Celine Larkin, Zelda Di Blasi, Ella Arensman

An affiliation for the last author is incorrectly omitted. In addition to institution number 1, Ella Arensman is also affiliated with the following institution: Department of Epidemiology & Public Health, University College Cork, Ireland.

