# Supplementary material for: Risk Factors for Repetition of Self-Harm: A Systematic Review of Prospective Hospital-Based Studies
Source: PLoS One. 2014 Jan 20;9(1):e84282. doi: 10.1371/journal.pone.0084282 (PMC3896350; doi:10.1371/journal.pone.0084282)
Supplement: Table S1 — Characteristics of Included Studies of Self-Harm Repetition. (DOCX) [file pone.0084282.s006.docx]

Table S1 (To be included online as Supporting Information)

*Characteristics of Included Studies of Deliberate Self-Harm (DSH) Repetition*

| Authors & Year | Level of intent | Method | Baseline N and Population [% male] | Setting and location | Follow-up (years) | Repetition detection (retention rate) | Circumstances of baseline data collection | Quality |
| --- | --- | --- | --- | --- | --- | --- | --- | --- |
| [Adam et al.,](#_ENREF_1) ([1983](#_ENREF_1)) | All | All | 98: Admitted for any intentional self-destructive act, however minor (32%) | A&E Dept, Christchurch Hospital | 1.5-2 | Interview (89%) | In hospital, 84% within 2 days | 2.5 |
| Aghanwa ([2004](#_ENREF_2)) | All | All except habitual wrist-cutting | 128: Suicide attempters, excludes non-suicidal wrist-cutting (31%) | Consultation- Liaison psychiatric service, Suva, Fiji | Max 2.5 | Records | Liaison psychiatry records | 2 |
| Allgulander & Fisher ([1990](#_ENREF_3)) | All | Psychoactive drug self-poisoning | 8895:Admissions of intentional self-poisoning with psychoactive drugs (38%) | Stockholm county, Sweden | <10 | Records | Records | 3.5 |
| Antretter et al.([2006](#_ENREF_4)) | All | All | 238: Presented with DSH (41.4%) | Pecs, Hungary and Hall, Austria | 2 | Records and interview | Interview and records | 4 |
| Archinard ([2000](#_ENREF_5)) | All | All | 22: Presented with suicide attempt (26%) | emergency ward of Geneva University Hospitals | 2 | Records | Interview | 1.5 |
| Bancroft et al ([1975](#_ENREF_6)) | All | All | 528: Presenting with DSH | General hospital, Oxford | 0.25-0.5 | Records | Records | 3 |
| Batt et al. ([1998](#_ENREF_7)) | All | All | 632: Admitted with DSH (37%) | University hospital, Rennes, France | 0.5 | Records | Psychiatric assessment | 3 |
| Beautrais ([2004](#_ENREF_8)) | All | “Medically serious” | 302: Medically serious DSH (45%) | Sole regional ED at Christchurch Hospital | 5 | Interview (81%) | Interview | 3.5 |
| Bergen et al.([2010](#_ENREF_9)) | All | All | 8030: Presenting with DSH (41.8%) | All hospitals in Oxford, Manchester and Derby | 2 | Records | Proforma | 4 |
| Bilén et al ([2010](#_ENREF_10)) | All | All | 1524: Presenting with DSH (35%) | General hospital Stockholm, Sweden. | 1 | Records | Records | 4 |
| Brittlebank et al.([1990](#_ENREF_11)) | All | All | 61:Presenting with DSH (39%) | District general hospital, Newcastle-upon-Tyne, UK. | 0.25-0.66 | Postal questionnaire (85%) | Postal questionnaire | 2.5 |
| Buglass & Horton ([1974a](#_ENREF_12), [1974b](#_ENREF_13)) | All | All | 2809: Admissions of parasuicide | Edinburgh Regional Poisoning Centre | 1 | Records | Records | 3 |
| Cailhol et al. ([2007](#_ENREF_14)) | All | All | 95:Suicide attempt, borderline personality disorder patients only (16%) | Emergency department, Geneva University Hospital | 1 | Records | Interview | 2 |
| Caldera et al.([2007](#_ENREF_15)) | All | All | 204: Cases of parasuicide living in the study area (31%) | Hospital admissions in Leon, Nicaragua | 2-5 | Self-report and records (52%) | Hospital interview usually within 24–48 hrs | 3.5 |
| Carter et al.([2002](#_ENREF_16)) | All | Self-poisoning and overdose | 1317: Presenting with deliberate self-poisoning (38%) | Hospital presentations in Newcastle, Australia | 1 | Records | Records | 3.5 |
| Carter et al.([1999](#_ENREF_17)) | All | Self-poisoning and overdose | 1241: Presenting with deliberate self-poisoning (35%) | Hospital presentations in Newcastle, Australia | <1 | Records | Records | 3.5 |
| Chandrasekaran & Gnanaselane ([2008](#_ENREF_18)) | All | All | 341: Presenting with DSH, with a score of greater than 25 on the Mini-Mental State Examination (45%) | Tertiary hospital in India | 2 | Interview (85.9%) | Interview in ward | 3.5 |
| Christiansen & Jensen ([2007](#_ENREF_19)) | All | All | 2614 (40%) | County of Funen, Denmark | Mean 2.88 | Records | Records | 4 |
| Colman ([2000](#_ENREF_20)); Colman et al ([2004](#_ENREF_21)) | All | All | 507: Presenting with DSH (33%) | All emergency departments in Edmonton, Alberta, Canada | 1-2 | Self-report; (83.6%) | Interview in home within 2 weeks | 4 |
| Cook & Anthony ([1999](#_ENREF_22)) | All | Overdose | 678: Intentional overdose of pharmaceutical substances (42%) | Emergency admissions unit in City of Coventry | 2 | Records | Records | 3.5 |
| Cooper et al ([2010](#_ENREF_25)) | All | All | 14997: Presented with DSH (42.1%) | Three general hospitals in Manchester, two in Derby and one in Oxford | 1 | Records | Records | 4 |
| Cooper et al.([2006](#_ENREF_23)) | All | All | 7185: Presented with DSH (44%) | All emergency departments in Manchester and Salford | 1 | Records | Records | 3 |
| Cooper et al.([2006](#_ENREF_24)) | All | All | 9086: Presented with DSH (44%) | All emergency departments in Manchester and Salford | 0.5 | Records | Records | 4 |
| Corcoran et al.([2004](#_ENREF_26)) | All | All | 1257: Presented with DSH (45%) | 10 emergency depts, 5 psychiatric hospitals and 3 prisons in south-west of Ireland | 1 | Records (53%) | Records | 4 |
| Corcoran et al.([1997](#_ENREF_27)) | All | All | 212: Presented with DSH | Psychiatry and general hospitals, Cork, Ireland | 0.5 | Records | Records | 3.5 |
| Courtet et al.([2004](#_ENREF_28)) | All | All except self-mutilation | 103: Hospitalized in the unit after a suicide attempt (17%) | Psychiatric unit, Montpellier, France | 1 | Interview (74%) | In hospital | 3 |
| Crawford & Wessely ([1998](#_ENREF_29)) | All | All | 308: Presented with DSH | 16 randomly selected general practices, Southwark, South London | <1.5 | Records | Records | 3 |
| De Leo et al.([2002](#_ENREF_30)) | All | All | 106: Suicidal behaviour, aged >60 years (37%) | Health facilities at nine sites across Europe | 1 | Interview (59%) | Interview | 1.5 |
| Dieserud et al. ([2003](#_ENREF_31)) | All | All | 50: Presented with DSH (34%) | Asker & Baerum: a local general hospital | 1.5 | Self-report (90%) and hospital records | Interview after discharge, mean 20.4 days after episode | 3.5 |
| Evans et al.([2000](#_ENREF_32)) | All | All | 467: Admitted following DSH and referred for psychiatric assessment (47% male) | Bristol Royal Infirmary | 1 | Self-report (90%) | Soon after admission | 4 |
| Garzotto et al.([1976](#_ENREF_33)) | All | All | 120: Psychiatric admissions following parasuicide | Psychiatric University Clinic of Verona | 1 | Self-report (76%) | Records | 1.5 |
| Géhin et al.([2009](#_ENREF_34)) | Confirmed intent | All | 65: Admissions for a suicide attempt (17%), aged 11-19 years | Children’s Hospital, Centre Hospitalo-Universitaire, Nancy, France | 10 | Survey (55%) and records (92%) | Records | 2.5 |
| Gibb et al.([2005](#_ENREF_35)) | All | All | 3690: Admitted for attempted suicide (40%) | Christchurch Hospital, New Zealand | <10 | Records | Records | 3.0 |
| Gilbody, House & Owens ([1997](#_ENREF_36)) | All | Self-poisoning and overdose | 1576: Attended with self-poisoning | Medical ward/A&E Leeds General Infirmary | 1 | Records | Records | 2.5 |
| Goldacre & Hawton ([1985](#_ENREF_37)) | All | Self-poisoning and overdose | 2492: Admitted for self-poisoning, aged 12 -20 years (26%) | Hospitals in Oxford region | 1-5 | Records | Records | 2.5 |
| Groholt et al.([2006](#_ENREF_38)) | Confirmed intent | “Medically serious” | 92: Admission following a suicide attempt, aged <18 years (10%) | Six medical wards in Oslo, Norway | 9 | Self-report (79%) | Unclear | 2.5 |
| Gunnell et al.([2002](#_ENREF_39)) | All | All | 381: Presented with DSH (42%) | A&E departments in Bristol and Bath | 1 | Records | Records | 3 |
| Harriss et al.([2005](#_ENREF_40)) | All | All | 2719: Presented with DSH (42%) | General hospital in Oxford | <8 | Records (91.5%) | Records | 4 |
| Hassanyeh et al. ([1989](#_ENREF_41)) | All | All | 98: Admitted with DSH | Newcastle-upon-Tyne | 1.5 | GP questionnaire (72.4%) | Interview | 2.5 |
| Haukka et al.([2008](#_ENREF_42)) | All | All | 18199: Admitted with an ICD diagnosis of attempted suicide (49%) | Finland | Mean 3.6 | Records | Records | 4 |
| Haw & Hawton ([2010](#_ENREF_44)) | All | All | 7856: Presented with DSH (38%) | General hospital in Oxford | 1 | Records | Records | 4 |
| Haw et al.([2007](#_ENREF_43)) | All | All | 4167: Presented with DSH (39%) | General hospital in Oxford | 3-10 | Records | Records | 4 |
| Haw et al.([2006](#_ENREF_45)) | All | All | 8368: Presented with DSH (42%) | General hospital in Oxford | 1 | Records | Records | 4 |
| Haw et al.([2003](#_ENREF_46)) | All | All | 150: Presented with DSH (39%) | General hospital in Oxford | 1-1.66 | Interview | Interview, 71% within a week | 2 |
| Hawton et al ([2012](#_ENREF_47)) | All | All | 5205: Presented with DSH, aged <18 years (25.5%) | Six general hospitals in Oxford, Manchester & Derby | 3-11 years | Records | Records | 3.5 |
| Hawton & Harriss ([2008](#_ENREF_54)) | All | All | 710: Presented with DSH, aged <15 years (16%) | General hospital in Oxford | 1.66-23 | Records | Hospital: records and self-report | 2.5 |
| Hawton & Harriss ([2006](#_ENREF_53)) | All | All | 730: Presented with DSH, aged >60 years (37.1%) | General hospital in Oxford | <23 | Records | Records | 2.5 |
| Hawton et al.([2003](#_ENREF_56)) | All | All (excluding repetitive minor self-injury) | 150: Presented with DSH, excluding repetitive minor self-injury, (39%) | General hospital in Oxford | 1-1.66 | Self-report (79%) | Interviews, 71% within 7 days | 1.5 |
| Hawton et al.([2002](#_ENREF_55)) | All | All (excluding repetitive minor self-cutting) | 146: Presented with DSH (39%) | General hospital in Oxford | 1-1.33 | Interview (80.8%) | Interview, 71% within a week | 2.5 |
| Hawton et al. ([1999](#_ENREF_57)) | All | Overdose | 45: Admitted with intentional self-poisoning, aged 12-18 years (16%) | General hospital in Oxford | 1 | Records | Interviewed within 24 hours | 3 |
| Hawton et al.([1997](#_ENREF_58)) | All | All | 724: Presented with DSH (38.7%) | General hospital in Oxford | 1 | Records | Records | 3 |
| Hawton & Fagg ([1995](#_ENREF_49)) | All | All | 1180: Presented with DSH (39%) | General hospital in Oxford | 1 | Records | Records | 3 |
| Hawton & Fagg ([1992](#_ENREF_48)) | All | All | 2282: Presented with DSH, aged10-19 years (27%) | General hospital in Oxford | 1 | Records | Records | 2.5 |
| Hawton et al.([1989](#_ENREF_51)) | All | All | 4371: Referred following suicide attempt (35.2%) | Emergency Psychiatric Services, general hospital in Oxford | 1 | Records | Records | 3 |
| Hawton et al. ([1988](#_ENREF_52)) | All | All | ?: Presented with DSH, females only | General hospital in Oxford | 1 | Records | Records | 2.5 |
| Hawton et al.([1980](#_ENREF_50)) | All | All | 1291: Admitted for DSH (31%) | General hospital in Oxford | 1 | Records | Records | 3 |
| Hepple & Quinton ([1997](#_ENREF_59)) | All | All | 100: Presenting with attempted suicide, aged > 65 years (36%) | General hospital in Oxford | Avg 3.5 | Records & interview (58%) | Records | 2.5 |
| Hjelmeland et al.([1998](#_ENREF_61)) | All | All | 552: WHO definition of DSH (37%) | Five Nordic centres | 1 | Records | Records | 4 |
| Hjelmeland ([1996](#_ENREF_60)) | All | All | 1016: Medically treated parasuicides, (40%) | All general and psychiatric hospitals, community health centres, and GPS in Sør-Trøndelag (county in Norway) | 1 | Records | Interview and records | 4 |
| Hultén et al.([2001](#_ENREF_62)) | All | All | 1264: Attempted suicide, aged 15-19 years (28%) | Emergency departments in hospitals in Padova, Helsinki, Oxford,Stockholm, Umeå ,Sør-Trøndelag, Würzburg | Mean 3.9 | Records | Records | 3.5 |
| Jakobsen et al ([2011](#_ENREF_63)) | All | All | 4170: attempted suicide aged 10-22 years | Hospitals in National Patient register, Denmark | Mean 4.5 | Records | Records | 4 |
| Johannessen et al ([2011](#_ENREF_64)) | All | All | 1304: Admitted after a suicide attempt (32%) | General hospital Asker , & municipal suicide prevention team Baerum | 0.5, 1, & 5 | Records | Records | 4 |
| Johannessen et al.([2009](#_ENREF_65)) | All | All | 330: Admitted after a suicide attempt (32%) | Local psychiatric hospital, Bærum, Norway | 1 | Records | Records | 4.5 |
| Johnston et al. ([2006](#_ENREF_66)) | All | All | 4743: Presenting with DSH (39%) | Three hospitals providing emergency care in the city of Manchester | 0.5 | Records | Records | 4 |
| Kapur et al. ([2006](#_ENREF_68)) | All | All | 9213: Presenting with DSH (43%) | 4 emergency departments in Manchester & Salford | 1 | Records | Records | 4 |
| Kapur et al.([2004](#_ENREF_67)) | All | Self-poisoning and overdose | 658: Presenting with intentional self-poisoning (44%) | 4 emergency departments in Manchester & Salford | 0.5 | Records | Records | 3 |
| Kapur et al.([2002](#_ENREF_69)) | All | Self-poisoning and overdose | 604: Presenting with intentional self-poisoning (45%) | Six general hospitals in north west England | 1 | Records | Records | 2.5 |
| Keeley et al.([2003](#_ENREF_70)) | All | All | 2287: Presenting with DSH (46%) | 10 emergency departments, 5 psychiatric hospitals & 3 prisons in Ireland | <2 | Records | Records | 4 |
| Kessel & McCulloch ([1966](#_ENREF_71)) | All | All | 511: Presenting with DSH | Emergency dept of Edinburgh Royal Infirmary | 1 | Records and self-report | Interview with patient and independent informant while hospitalised | 3.5 |
| Kreitman & Foster ([1991](#_ENREF_72)) | All | All | 2813: Admitted for DSH (42%) | Emergency dept of Edinburgh Royal Infirmary | <1 | Records | Records | 4 |
| Laurent et al.([1998](#_ENREF_73)) | All | All | 587: Admitted following a suicide attempt, aged <18 years, 27% male | General/emergency paediatric wards, paedopsychiatry, clinical toxicology services at University Hospital of Grenoble, France | Mean 5.3 | Survey patients & GP (48%) | Records | 2 |
| Lebret et al.([2006](#_ENREF_74)) | SA? | All | 59: Admitted following a suicide attempt, aged > 60 years (41%) | Hospital psychiatric service, Clermont Ferrand, France | 3-10 | Letter and phone call to attending physician; (86%) | Records | 3.5 |
| Lee et al ([2012](#_ENREF_75)) | Confirmed/apparent intent | All | 145: suicide attempts (32%) | Medical centre, Taiwan | 1 | Interview | Interview |  |
| Lilley et al.([2008](#_ENREF_76)) | All | All | 7344: Presenting with DSH (44%) | All six EDs in Oxford, Manchester and Leeds | <1.5 | Records | Records | 3 |
| Links et al (2012) | Confirmed | All | Presenting with ideation (18%) or attempt () | Inpatient psychiatric service and a short-stay crisis stabilization unit, Toronto, Canada | 0.5 | Interview | Interview | 3.5 |
| Mackay ([1979](#_ENREF_78)) | All | Self-poisoning and overdose | 3733: Presenting with intentional self-poisoning (36%) | Emergency department of Glasgow Western Infirmary | 1 | Records | Records | 1.5 |
| Mayo ([1974](#_ENREF_79)) |  |  | 111: Admitted for drug overdose | New York | >1 | Interview (87.4% ) | Interview | 1 |
| McAuliffe et al.([2008](#_ENREF_80)) | All | All | 152: Presenting with DSH (38%) | One emergency department in Cork Ireland | 1 | Hospital records | Interview, usually within 2 days | 3.5 |
| McEvedy ([1997](#_ENREF_81)) | All | Self-poisoning and overdose | 705: Admitted for intentional self-poisoning | Charing Cross  Hospital, London | <4 | Records | Records | 2.5 |
| Méan et al.([2005](#_ENREF_82)) | Unclear | All plus ideation only (36.6%) | 186: Admitted following suicide attempt/overwhelming suicidal ideation, aged 16-21 years (20%) | Two university hospitals’ EDs, Lausanne and Geneva | 0.5 & 1.5 | Self-report (79.6%) | Interview | 3 |
| Mehlum et al.([2010](#_ENREF_83)) | All | All | 911: Admitted with suicide attempt (34.8%) | Aker University Hospital | <10 | Records | Proforma | 4 |
| Monnin et al ([2012](#_ENREF_84)) | Confirmed intent | All | 273: Admitted with suicide attempt | Psychiatric emergency units of University Hospital of Besançon & Hospital of Dole, France | 2 | Records | Proforma | 4 |
| Morgan et al.([1976](#_ENREF_85)) | All | All | 279: Presenting with DSH (37%) | Bristol Royal Infirmary Emergency Department | 1-2, | Self-report (73%), relative-report, health/community worker report, & hospital notes (97%) | Interview | 3.5 |
| Morton ([1993](#_ENREF_86)) | All | All | 691: Admitted with DSH, economically active males only | Regional Poisoning Treatment Centre, Edinburgh | 1 | Records | Records | 3.5 |
| Murphy et al ([2012](#_ENREF_87)) | All | All | 1177: Presented with DSH, aged 60+ (44%) | Six general hospitals in Oxford, Manchester & Derby | 1 | Records | Records | 3.5 |
| Myers et al.([1988](#_ENREF_88)) | All | All | 365: Admitted with DSH (39.5%) | Accident dept of North Staffordshire Hospital Centre | 1 | Records | Records | 3 |
| Nordentoft & Branner ([2008](#_ENREF_89)) | All | All | 351: Presenting after suicide attempt, aged 18-45 years, (30.5%) | Copenhagen Suicide Prevention Centre | 1 | National Patient Register | Interview | 2.5 |
| Öjehagen et al.([1992](#_ENREF_90)) | All | Self-poisoning and overdose | 79: Admitted after intentional self-poisoning (42%) | Medical intensive care unit, University hospital Lund | 1 | Interview (74.7%) | Interview | 1.5 |
| Owens et al.([1994](#_ENREF_91)) | All | Self-poisoning and overdose | 992: Presenting with intentional self-poisoning (40%) | Emergency department, Nottingham, England | 1 | Records | Records | 2.5 |
| Payne et al.([2009](#_ENREF_92)) | All | Self-poisoning and overdose | 50891: Admitted with intentional self-poisoning (43%) | All Scottish hospitals | 2 | Records | Records | 3.5 |
| Perry et al ([2012](#_ENREF_93)) | All | All | 48,206: presented with DSH | All emergency departments in Ireland | <7 | Records | Records |  |
| Peterson & Bongar ([1990](#_ENREF_94)) | Unclear | All plus ideation only | 616: Presenting with suicidal behaviour and ideation, (52.4%) | University of Massachusetts Emergency Mental Health Service | <1 | Records | Records | 3 |
| Petrie & Brook,([1992](#_ENREF_95)) | All | All | 150: Admitted with DSH | Large New Zealand general hospital | 0.5 | Postal questionnaire (76%) and records | Computer interview in hospital | 4 |
| Petrie et al.([1988](#_ENREF_96)) | All | All | 67: Attempted suicide, (27%) | Three general hospitals in New Zealand | 0.5 | Postal questionnaire (69%) | Survey in hospital | 3 |
| Pino et al. ([1979](#_ENREF_97)) | All | Overdose | 100: Intentional overdose (31%) | Clinical toxicology unit, Munich | 6 | Interview (66%) | Interview | 1.5 |
| Randall et al ([2012](#_ENREF_98)) | All | All plus ideation only (55.5%) | 157: Presented with self-harm or suicidal ideation (55.5% ideation only) (51.1% male) | 2 EDs in Edmonton, Canada | 0.25 | Phonecall (82%) and records | Structured interview and chart review | 4 |
| Ruiz-Doblado ([2001](#_ENREF_99)) | All | All | 138: Suicide attempts | Osuna Hospital, Seville, Spain. | Not spec | Records | Records | 2.5 |
| Sakinofsky & Roberts ([1990](#_ENREF_100)) | All | All | 228: Presenting with DSH (35%) | Four major general hospitals in Hamilton, Canada | 0.25 | Self-report (82%) | Interviewed within 3 days or post-detoxification | 4 |
| Santos et al ([2009](#_ENREF_101)) | All | All | 34: Presented with self-harm, 15-24 years of age (17.7%) | Emergency department in Coimbra, Portugal | 0.75 | Self-report | Interviewed a week after presentation | 0.5 |
| Sathianathan & Sadowski ([1996](#_ENREF_102)) | All | All | 354: Attempted suicide for first time | One general hospital, Madras, India | 1 | Self-report (76%) | Unclear | 4 |
| Scoliers et al, ([2009](#_ENREF_103)) | All | All | 874: Presenting with DSH (34%) | Emergency department of University Hospital Ghent | 5 | Interview (41.3%) | Interview | 3.5 |
| Scott et al. ([1997](#_ENREF_104)) | All | Overdose | 43: Admitted with intentional self-poisoning, with a score of least 1 on the Buglass and Horton scale and no reported previous DSH (27.2%) | Newcastle-upon-Tyne | 0.25 | Records | Interview | 2 |
| Sellar et al.([1990](#_ENREF_105)) | All | Self-poisoning and overdose | 3034: Admitted for intentional self-poisoning, aged 12-20 years (29%) | General hospitals in Oxford region | 1-6 | Records | Records | 2.5 |
| Sertöz ([2010](#_ENREF_106)) | Unclear | All | 55: Admitted for attempted suicide | Ege University Medical Emergency Department, Turkey | 5.4 | Interview (78%) | Interview | 3 |
| Siani et al. ([1979](#_ENREF_107)) | All | All | 147: First-ever admitted for DSH | Psychiatric university clinic of Verona | 1 | Self-report (91.2%) | Hospital interview | 2.5 |
| Sidley et al.([1999](#_ENREF_108)) | All | Overdose | 66: Admitted following intentional drug overdose, “High-risk” patients (5 or more of 11 items in Kreitman and Foster’s scale) (55%) | Emergency Department of North Manchester General Hospital | <1 | Hospital records and self-report | Interview mean 3.1 days | 3.5 |
| Sinclair et al.([2010](#_ENREF_110)) | All | All | 150: Presented with DSH (38%) | General hospital in Oxford | 6 | Self-report (67%) & hospital records | Interview, majority within a week | 2.5 |
| Sinclair et al.([2007](#_ENREF_109)) | All | All | 150: Presented with DSH (29%) | General hospital in Oxford | 7 | Interview (48.7%): only “recent” repetition | Interview, 69% within a week | 3 |
| Sjöström et al ([2012](#_ENREF_111)) | Confirmed/apparent intent | All | 165: Admitted after suicide attempt (22%) | Medical & psychiatric wards at Sahlgrenska University Hospital, Göteborg, Sweden | 3 | Records | Within a week | 3.5 |
| Sjöström et al.([2009](#_ENREF_112)) | Confirmed/apparent intent | All | 165: Admitted after suicide attempt (22%) | Medical & psychiatric wards at Sahlgrenska University Hospital, Göteborg, Sweden | 2 | Records | Within a week | 3.5 |
| Söderberg et al.([2004](#_ENREF_113)) | All | All | 64: Admitted after parasuicide (37%) | Somatic & psychiatric wards, Umeå University Hospital, Sweden | Mean 7.5 | Interview (79.7%) | Interview | 3.5 |
| Spirito et al.([1994](#_ENREF_114)) | All | All | 62: Presented with act they described as suicide attempt, regardless of lethality | Regional trauma centre emergency department in Rhode Island | 0.25 | Telephone questionnaire with parents & participants (79.5%) | Questionnaires in emergency dept | 2.5 |
| Steeg et al ([2012](#_ENREF_115)) | All | All | 29571 episodes: Presented with DSH (40.6%) | Six general hospitals in Oxford, Manchester & Derby | 0.5 | Records | Records | 4 |
| Stenager et al.([1994](#_ENREF_116)) | All | All | 72: Admitted following suicide attempt (35%) | Dept of Psychiatry, Odense University Hospital, Denmark | 1 | Records | Interview, majority within a week | 3 |
| Suleiman et al.([1989](#_ENREF_117)) | All | All | 92: Presenting with DSH (14.1%) | General hospital in Kuwait | <2 | Records and interview (89.1% retention) | Interview | 2.5 |
| Taylor et al.([1998](#_ENREF_118)) | All | Overdose | 381: Presenting with intentional overdose (32.8%) | Emergency dept of Geelong Hospital, Victoria, Australia | <2 | Records | Records | 2.5 |
| Tejedor et al.([1999](#_ENREF_119)) | All | All | 150: Admitted after suicidal act (44%) | Psychiatric Department of Santa Cruz y San Pablo Hospital, Barcelona, Spain | Mean 10 | Self-report (97%) | Interview | 3.5 |
| Träskman-Bendz et al.([1992](#_ENREF_120)) | Confirmed | All | 61: Admitted after suicide attempt, with intent to die | Research ward, Sweden | 1 | Unclear | Interview avg 16 days after admission | 2 |
| Vajda & Steinbeck ([2000](#_ENREF_121)) | Confirmed | All | 112: Admitted after intentional harm with some wish to end life, aged 13-20 (32%) | Emergency department at Royal Prince Alfred Hospital, New South Wales, Australia | 1 | Records | Records | 3 |
| van Aalst et al.([1992](#_ENREF_122)) | Confirmed | Violent self-injury | 118: Admitted with violent suicide attempt , serious intent to die (83%) | Dept of Surgery, Vanderbilt University Medical Centre, Tennessee | 1-6 | Self-report/family-report (88%) | Records and retrospective self-report about conditions around index episode | 2 |
| van Egmond et al.([1993](#_ENREF_123)) | Unclear | All | 158: Admitted after “suicide attempt”, Aged >20 years, female only | University hospitals of Leiden & Utrecht | 1 | Self-report (80%) | Interview within 8 wks of discharge | 2 |
| Verkes et al.([1997](#_ENREF_124)) | Unclear | All | 106: Presenting after “attempting suicide” with history of at least one previous suicide attempt (39%) | Emergency departments of Leiden & Rotterdam University Hospitals | 1 | Unclear | Interview, majority within two weeks | 3 |
| Waern et al.([2010](#_ENREF_125)) | Confirmed/apparent intent |  | 165: Admitted after suicide attempt (22%) | Emergency wards at Sahlgrenska University Hospital, Göteborg, Sweden | 3 | Records | Interview, majority within 3 days | 3.5 |
| Wang & Mortensen ([2006](#_ENREF_126)) | All | All | 125: Presenting with DSH (46% male) | Emergency department, Faroe Islands | 20-41 | Records | Records | 3.5 |
| Wang et al.([1985](#_ENREF_127)) | Unclear | All | 99: Admitted with suicide attempt (52%) | Dept of Psychiatry, Odense | <3.4 | Records | Interview | 3.5 |
| Wiktorsson et al. ([2011](#_ENREF_128)) | Confirmed/apparent intent | All | 101: Admitted with suicide attempt, aged 70 yrs+ (45%) | Emergency departments at five hospitals in western Sweden | 1 | Records & self-report | Interview (within median 11 days) | 2.5 |
| Wilkinson & Smeeton ([1987](#_ENREF_129)) | All | All | 1376: Admitted with DSH (38.5%) | Edinburgh Regional Poisoning Treatment Centre | 1-2 | Records | Records | 3 |
| Yeo & Yeo ([1993](#_ENREF_130)) | All | All | 178: Presenting with DSH (38%) | Emergency dept, Royal Hallamshire Hospital, Sheffield | 0.5 | Records | Records | 2.5 |
| Yip et al ([2011](#_ENREF_131)) | All | All | 90: Presenting with DSH (29%) | Pamela Youde Nethersole Eastern Hospital, Hong Kong | 0.5 | Records | Records | 2.5 |
| Zahl & Hawton ([2004](#_ENREF_132)) | All | All | 11583: Presenting with DSH (40%) | General hospital in Oxford | Avg 11.4 | Records | Records | 2 |
